# Isolation, characterisation, and genome sequencing of *Rhodococcus equi*: a novel strain producing chitin deacetylase

**DOI:** 10.1038/s41598-020-61349-9

**Published:** 2020-03-09

**Authors:** Qinyuan Ma, Xiuzhen Gao, Xinyu Bi, Linna Tu, Menglei Xia, Yanbing Shen, Min Wang

**Affiliations:** 1 Key Laboratory of Industrial Fermentation Microbiology (Tianjin University of Science & Technology), Ministry of Education, Tianjin Key Lab of Industrial Microbiology, College of Biotechnology, Tianjin University of Science and Technology, Tianjin, 300457 P.R. China; 2 School of Life Science, Shandong University of Technology, Zibo, 255049 China

**Keywords:** Carbohydrates, Biopolymers, Applied microbiology, Bacteria

## Abstract

Chitin deacetylase (CDA) can hydrolyse the acetamido group of chitin polymers to produce chitosans, which are used in various fields including the biomedical and pharmaceutical industries, food production, agriculture, and water treatment. CDA represents a more environmentally-friendly and easier to control alternative to the chemical methods currently utilised to produce chitosans from chitin; however, the majority of identified CDAs display activity toward low-molecular-weight oligomers and are essentially inactive toward polymeric chitin or chitosans. Therefore, it is important to identify novel CDAs with activity toward polymeric chitin and chitosans. In this study, we isolated the bacterium *Rhodococcus equi* F6 from a soil sample and showed that it expresses a novel CDA (ReCDA), whose activity toward 4-nitroacetanilide reached 19.20 U/mL/h during fermentation and was able to deacetylate polymeric chitin, colloidal chitin, glycol-chitin, and chitosan. Whole genome sequencing revealed that ReCDA is unique to the *R. equi* F6 genome, while phylogenetic analysis indicated that ReCDA is evolutionarily distant from other CDAs. In conclusion, ReCDA isolated from the *R. equi* F6 strain expands the known repertoire of CDAs and could be used to deacetylate polymeric chitosans and chitin in industrial applications.

## Introduction

Chitin is the second most abundant biopolymer after cellulose and is mainly obtained as a waste product of the seafood industry at a relatively low cost^1^. The chitin derivative, chitosan, is a linear polysaccharide comprised of β-(1→4)-linked glucosamine and N-acetyl glucosamine units which are randomly arranged within the chitosan polysaccharide chain^2^. Due to its ability to dissolve in dilute acids, chitosan is more useful than its crystalline precursor chitin in various industrial applications^3^, including the biomedical and pharmaceutical industries, food production, agriculture, and water treatment^4^.

Chitosan occurs naturally and is mainly found in the cell walls of certain fungi, the exoskeletons of certain insects (such as the abdominal wall of termite queens), and in some yeasts^5,6^. Although chitosan can be extracted from fungal sources^7^, the method is commercially inapplicable as it provides too low a yield at too high a cost. Therefore, chitosan is still obtained by treating marine-derived chitin with thermo-alkaline^8^, a method that is inexpensive and results in high yields but is environmentally unsafe and difficult to control, producing a heterogeneous range of products depending on the degree of deacetylation^9^. The enzymatic production of chitosan using microbial chitin deacetylase (CDA; EC 3.5.1.41), which can hydrolyse the acetamido group in chitin polymers to produce chitosans^10^, is an environmentally-friendly process that is easy to control and results in a high yield of homogeneous end products^11^.

CDAs have been identified in marine and soil bacteria, several fungi, a few insects, and at least one virus^12,13^. Fungal CDAs exist mostly as *N*-glycosylated (20–70%) glycoproteins, while bacterial CDAs are mainly chitin oligosaccharide deacetylases (CODs) active toward low molecular weight CODs^1^; however, some bacterial CDAs also show broad substrate specificity^14^. Only a few microbial strains are known to produce CDAs, and they require specialised fermentation conditions for CDA production^15^. Since, CDAs have not yet been industrialised, the identification of a bacterial CDA with high activity toward polymeric chitin and chitosans would provide a straightforward approach for enzymatically converting chitin into chitosan.

In this study, we collected soil from different environmental sources and screened its CDA activity, identifying a strain producing high levels of CDA which was characterised by whole genome sequencing and designated as *Rhodococcus equi* F6. We then examined the CDA-producing capacity of *R. equi* F6 during fermentation and evaluated the activity of crude CDA isolated from *R. equi* F6 (ReCDA) toward polymeric chitin and chitosans.

## Results and Discussion

### Novel chitin-deacetylase-producing bacterial strain identified from soil

More than 100 soil samples were collected from different regions of China, including Chengdu, Shenyang, Xi’an, and numerous cities in Shandong province. The samples were selected randomly to obtain a wide range of strains. The primary screen determined the deacetylation activity of microorganisms present in each soil sample using a plate-based enzymatic assay with 4-nitroacetanilide as the colour indicator. After 2–3 days of incubation, colonies with a yellow circle indicating the occurrence of deacetylation were selected for secondary screening, during which their enzymatic activity was determined following fermentation in LB medium. The F6 strain exhibited the greatest CDA activity of the 16 positive isolates (Supplementary Table S1); therefore, this strain was selected for further taxonomic, physiological, and biochemical analyses.

### Phylogenetic classification and characteristics of the novel CDA-producing strain F6

**Figure 1:**
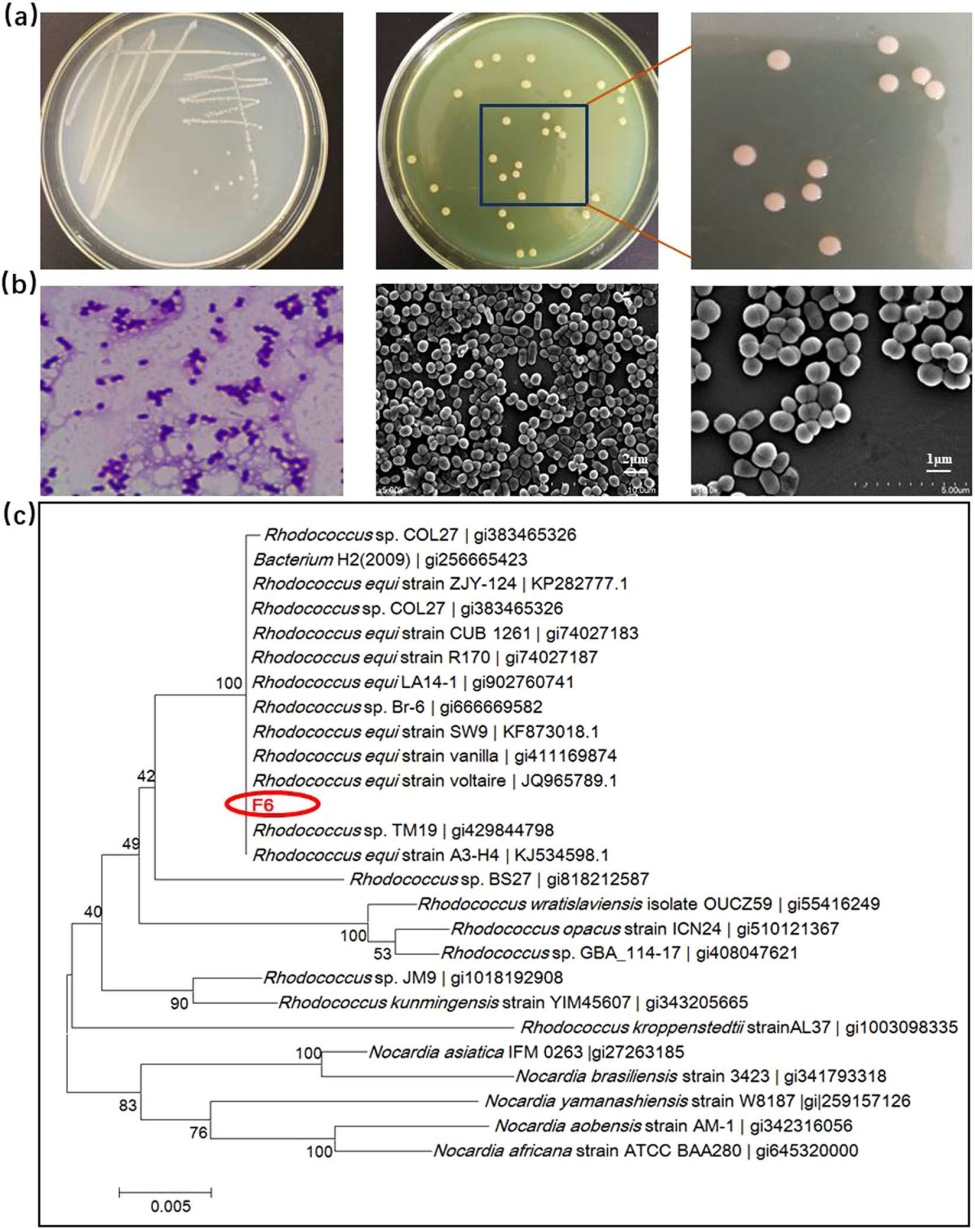
Morphological and phylogenetic characteristics of the F6 strain. (**a**) Colony morphology of the F6 strain. (**b**) Gram staining and SEM image showing the coccoid shape of the F6 strain. (**c**) Phylogenetic analysis of the F6 strain based on BLAST results.

When incubated for 2–3 days at 37 °C, colonies of the F6 strain appeared light pink, round, smooth, opaque, glistening, and mucoid (Fig. 1a). In addition, we found that the F6 strain was gram-positive (Fig. 1b), non-motile, aerobic, and did not produce spores. Scanning electron microscopy (SEM) images revealed that the F6 strain was coccoid in shape, with a diameter of 0.5–1.0 μm (Fig. 1b). Biolog-based identification assays indicated that the F6 strain utilised 15 different carbon sources, while the strain also grew in the presence of acetic acid, which is the by-product of CDA-catalysed deacetylation. The phenotypic and chemotaxonomic characteristics of the F6 strain are summarised in Table 1.

**Table 1:** Phenotypic and chemotaxonomic characteristics of *R. equi* F6.

Characteristic	Reaction	Characteristic	Reaction
Colonies:	Light pink, opaque, smooth	Cells:	Distinct cocci
pH:	4.0–9.0	Temperature:	25–40 °C
**Utilisation as sole carbon and energy source:**			
D-Ribose	+	Xylitol	−
D-Fructose	−	Acetic Acid	+
D-Glucose	+	α-Hydroxybutyric Acid	+
Sucrose	−	β-Hydroxybutyric Acid	+
D-Mannitol	−	α-Ketovaleric Acid	+
Dextrin	+	D-Cellobiose	−
D-Arabinose	−	D-Sorbitol	−
D-Xylose	+	L-Malic Acid	+
D-Galactose	−	Pyruvic Acid Methyl Ester	+
L-Rhamnose	−	Succinic Acid Mono-Methyl Ester	+
Lactamide	+	Propionic Acid	+
D-Lactic Acid Methyl Ester	+	Pyruvic Acid	+
L-Lactic Acid	+	Tween 40	+
D-Turanose	−	Tween 80	+
Arabitol	−	Lactose	−
myo-Inositol	−	Maltose	−
Inulin	−	Melezitose	−
N-Acetylglucosamine	−	Glycerol	+

+, Positive; −, negative.

Based on the results of the physiological, biochemical, and 16S rDNA sequence analyses (Fig. 1c), the F6 strain was classified as belonging to the genus *Rhodococcus*. Further alignment of the 16S rDNA of the F6 strain with other 16S rDNA sequences in the GenBank database revealed that the strain had 100% identity with several *Rhodococcus equi* strains. Therefore, the F6 strain was designated as *Rhodococcus equi* F6 (*R. equi* F6) and deposited into the China General Microbiological Culture Collection as *Rhodococcus equi* under the accession number 14861. However, as shown in Supplementary Table S2, the biochemical and physiological characteristics of *R. equi* F6 were not always consistent with those of previously described *Rhodococcus* strains^16–21^, suggesting that *R. equi* F6 may be a novel strain.

*Rhodococcus* species are known to biodegrade compounds that are not easily degraded by other organisms^22–24^, making them promising biocatalysts for industrial applications. In addition, members of the *Rhodococcus* genus can degrade natural hydrophobic compounds and xenobiotics, thus have been used in environmental, pharmaceutical, and chemical fields in addition to energy^25,26^. To the best of our knowledge, this is the first study to report CDA production by a novel *R. equi* strain.

### CDA production capacity of *R. equi F6*

**Figure 2:**
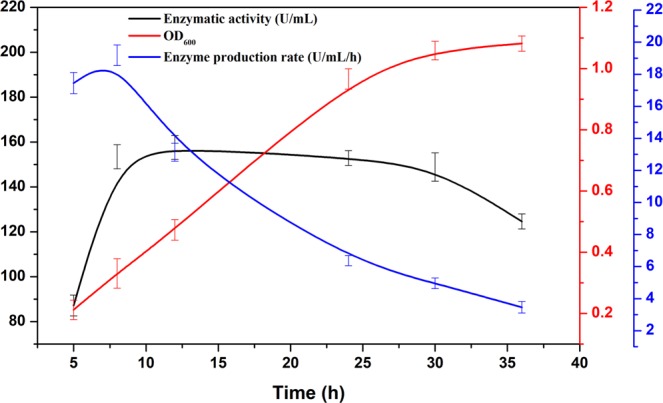
Cell growth and CDA production curves for *R. equi* F6.

Since the CDA produced by *R. equi* F6 (ReCDA) was localised intracellularly, we investigated the kinetics of ReCDA production by sampling the fermentation broth every few hours and determining enzymatic activity. The kinetics of *R. equi* F6 cell growth and CDA production in the main culture are shown in Fig. 2. CDA activity reached 87.2 U/mL during the first 5 h and then increased to the maximum level of 157.6 U/mL after 12 h. Moreover, CDA production by *R. equi* F6 exceeded that previously reported in other microorganisms^27–29^, which could lower the cost of recycling fermenters and bulk production. Biolog-based analysis indicated that the F6 strain did not have complex fermentation requirements for CDA production, making it suitable for industrial applications, while further mutation breeding of *R. equi* F6 could produce a strain with even higher CDA yields.

### Deacetylation of polymeric chitosans and chitin by crude *ReCDA*

We measured acetic acid production by HPLC analysis to assess whether crude ReCDA could hydrolyse different polymeric substrates. While crude ReCDA was able to hydrolyse colloidal chitin, glycol-chitin, and chitosan, it had no effect on water-insoluble chitin (Supplementary Fig. S1a). ReCDA hydrolysis was further characterised by MALDI-TOF MS, finding that the average molecular weight of glycol-chitin decreased significantly after 6 h, resulting in a mixture of compounds with different degrees of deacetylation (DD) (Supplementary Fig. S1b).

The majority of known bacterial CDAs are active only toward low-molecular-mass oligomers^1^; for instance *Vibrionaceae* CDA can only deacetylate substrates with degrees of polymerisation (DP) of 2–6^30^, while the activity of *Shewanella* CDA decreases from DP2 to DP4, with no activity at DP5^31^. ReCDA displays several advantages over these other bacterial CDAs since it can hydrolyse polymeric substrates and has high activity toward other substrates, as shown in Table 2. Moreover, ReCDA retained its enzymatic activity at high temperatures, low pH, and in presence of most divalent cations (Supplementary Fig. S2). Although we used a crude ReCDA preparation instead of the pure enzyme in this study, it is worth noting that crude enzymes are often used directly in industry^32^; thus, ReCDA could be used to prepare chitosan in combination with partial chemical treatment or dissolving with pretreated chitin, which will be reported in the near future. Consequently, we will purify and functionally characterise ReCDA in our future study.

**Table 2:** Relative activity of crude ReCDA toward different substrates.

Substrate	Peak area for acetate (mAU*s)	Relative activity (%)^a^
4-Nitroacetanilide	71.36 ± 1.09	100.00
N-Acetyl-DL-methionine	139.72 ± 2.85	204.12 ± 1.49
Chitosan (DAs, 85%)	92.99 ± 1.05	132.94 ± 0.93
N-acetylglucosamine	91.40 ± 1.42	130.52 ± 0.90
Colloidal chitin	78.73 ± 2.78	111.20 ± 2.40
Glycol-chitin	64.23 ± 2.01	89.12 ± 1.72
N-Acetyl-DL-tryptophan	63.44 ± 1.65	88.36 ± 1.57
N-Acetyl-L-leucine	46.74 ± 1.58	62.32 ± 1.91
N -Acetyl-L-cysteine	44.70 ± 1.97	59.36 ± 2.03
3-Acetylindole	34.98 ± 1.46	44.19 ± 1.92
Beta-D-Ribofuranose 1-acetate 2,3,5-tribenzoate	24.54 ± 1.26	28.56 ± 2.06
Chitooligosaccharides (2–6)	21.38 ± 1.51	23.85 ± 1.97
Powdered chitin	0.00	0.00

^a^Relative activity was determined by HPLC analysis and activity with 4-nitroacetanilide as the substrate was used as the standard.

### Whole genome sequencing, assembly, and annotation of R. *equi F6*

**Figure 3:**
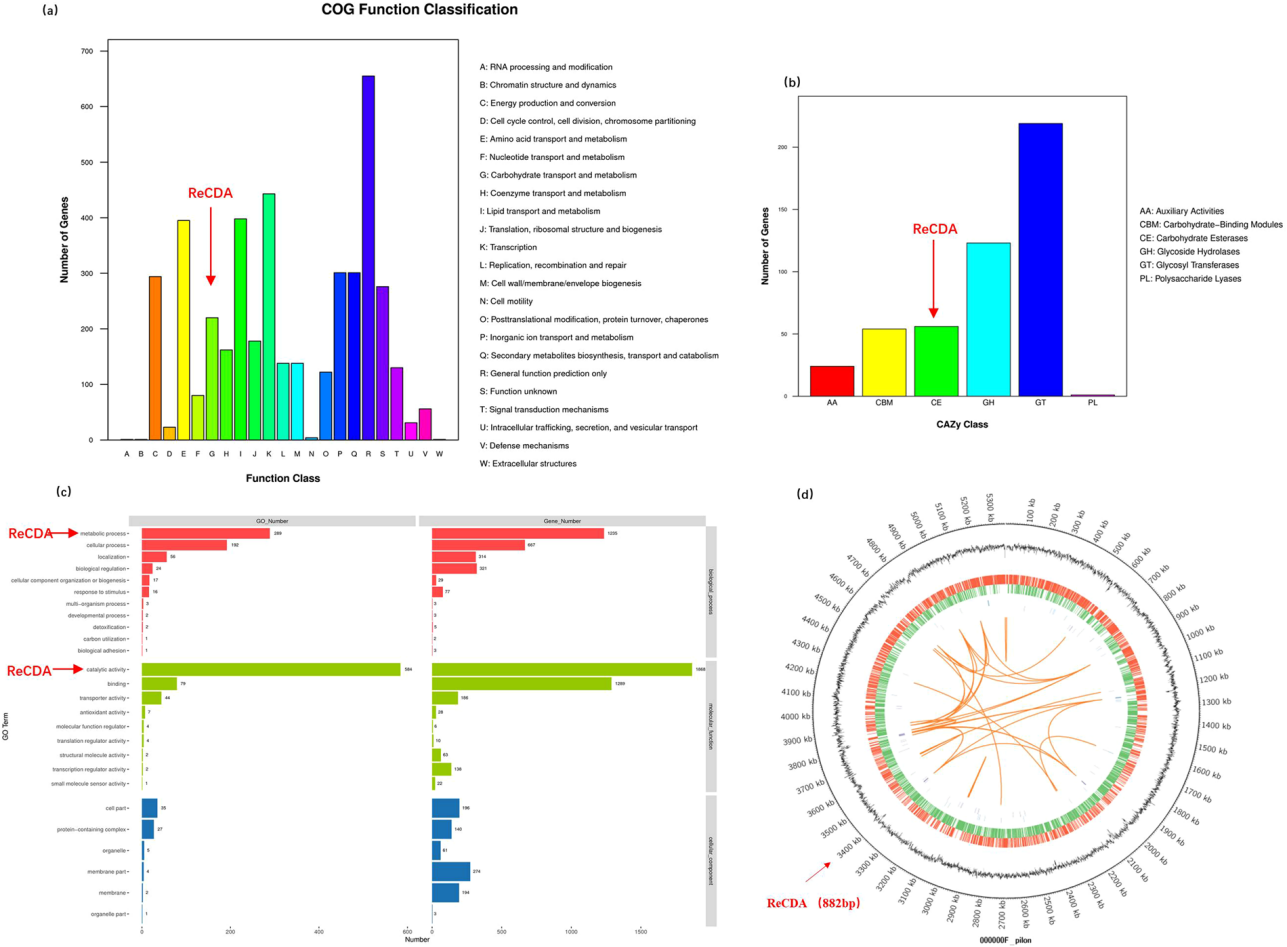
Whole genome analysis of *R. equi* F6. (**a**) COG functional gene classification. Abscissa represents COG functional classification and ordinate represents the number of genes annotated within each classification. (**b**) CAZy family distribution map. Abscissa represents CAZy family classification and ordinate represents the number of genes. (**c**) Histogram showing the distribution of Gene Ontology (GO) terms. Abscissa represents the gene number and ordinate represents GO terms. Different colours are used to distinguish biological processes, cellular components, and molecular functions. (**d**) Genomic map. From outside to inside: first circle shows genome location information; second circle shows GC content; third circle (red) shows genes encoded on positive strand; fourth circle (green) shows genes encoded on negative strand; fifth circle (blue) shows ncRNAs on positive strand; sixth circle (purple) shows ncRNAs on negative strand; and seventh circle (orange) shows long genome segment repetitive sequences.

To identify genes involved in chitin deacetylation, we performed whole genome sequencing on *R. equi* F6, with *de-novo* genome assembly revealing that the *R. equi* F6 genome is 5,354,717 bp in length, has a GC content of 68.62%, and contains 4,996 coding sequences and 126 total ncRNAs. CAZy annotation was successfully performed on 477 protein encoding genes (PEGs), 4,348 COG genes, 1,404 GO entries, and 3,679 Kyoto Encyclopaedia of Genes and Genomes (KEGG) pathways. Functional PEG annotation revealed a total of 23 classifications, with most predicted to be involved in general functions such as transcription, lipid transport, metabolism, amino acid transport, and metabolism. A total of 220 genes were involved in carbohydrate transport and metabolism (including ReCDA), representing 5.06% of the total number of genes (Fig. 3a), while the 477 genes identified using CAZy represented 10.97% of the total number of genes, indicating that *R. equi* F6 can metabolise carbohydrates^33^. Next, we performed PEG analysis with Blastp to identify CAZymes encoded by ReCDA (Fig. 3b) and annotated ReCDA using CE 4. A histogram of target gene distribution using GO terms is shown in Fig. 3c. ReCDA was annotated for biological processes (carbohydrate metabolic processes) and molecular functions (catalytic and hydrolase activity toward carbon-nitrogen, but not peptide, bonds), and its genetic sequence was annotated uniquely in the whole genome database, suggesting that it is the only chitin deacetylase in *R. equi* F6. The ReCDA gene (Supplementary Data 5) was 882 bp in length and located at 3,400 kb in the genomic map (Fig. 3d).

To further explore whether *R. equi* F6 could be used to degrade chitin, we analysed its whole genome for the presence of enzymes involved in chitin deacetylation and hydrolysis. A total of 51 genes were annotated as deacetylases or chitinases (Supplementary Table S3), accounting for 10.69% of the 477 genes annotated in the CAZy family. In addition to the single *ReCDA* gene, four genes encoding N-acetylglucosamine deacetylases and 16 encoding diacetylchitobiose deacetylases were annotated within the 23 deacetylases, indicating that *R. equi* F6 is a potent chitin decomposer. Notably, only five bacterial CDAs have been reported to date^13,14,31,34^.

### Phylogenetic analysis for *ReCDA*

**Figure 4:**
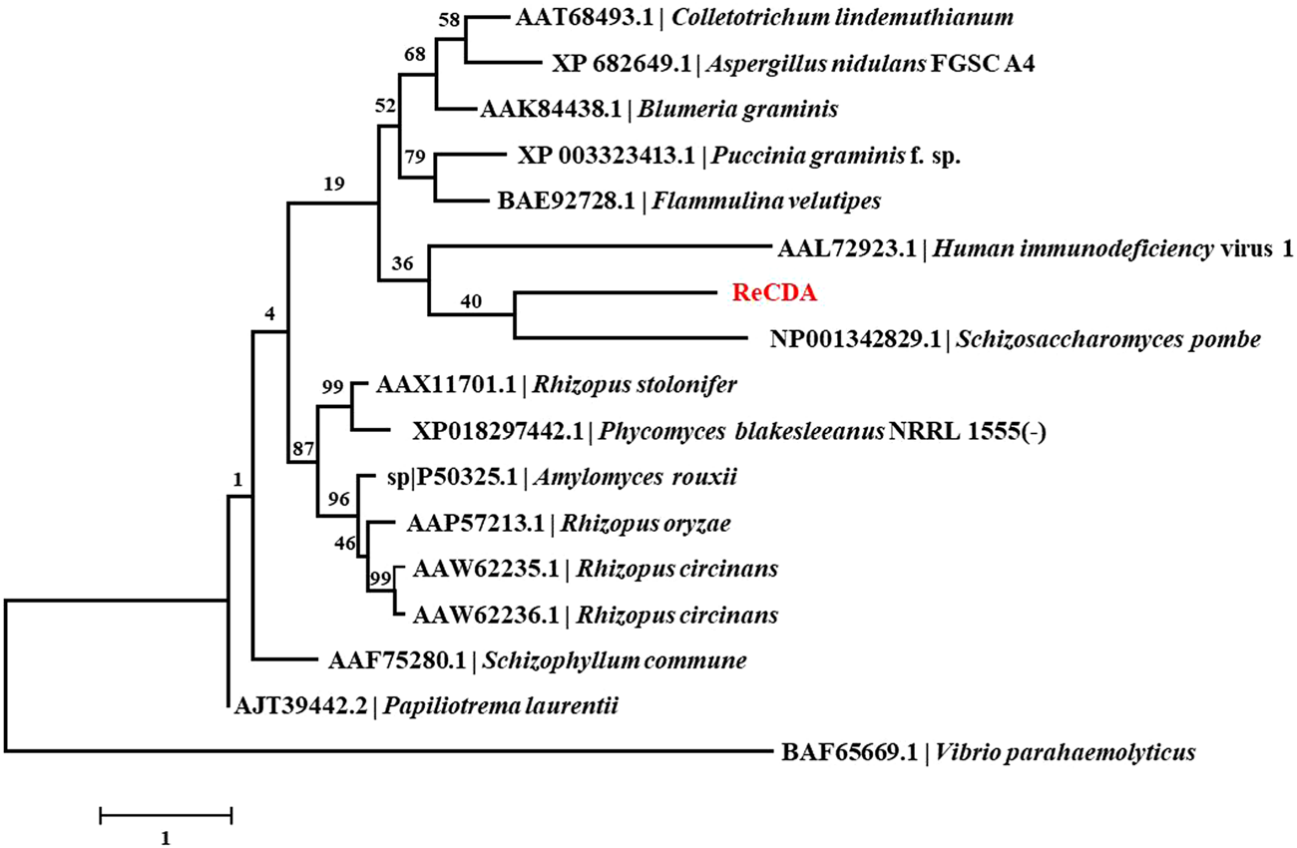
Molecular phylogenetic analysis of CDAs from *R. equi* F6 and several other known bacteria. Evolutionary history was inferred using the maximum likelihood method based on a JTT matrix-based model^52^. The amino acid sequences used in this tree were reported in^37–47^. Clustal-X2 (Version 1.83) was used for multiple-sequence alignment.

Finally, we used phylogenetic analysis to compare ReCDA with known CDAs from other microorganisms. ReCDA was located in the same node as the CDA from fission yeast *Schizosaccharomyces pombe* (NCBI Reference Sequence: NP_001342829.1; Fig. 4), with multiple-sequence alignments indicating that ReCDA has 87% query coverage and 21.77% identity with *S. pombe* CDA. The identity of ReCDA with other known CDAs was below 32%, while the majority of the bootstrap values were below 60%, indicating that the node was not well supported. Taken together, these low degrees of homology indicate that ReCDA is a novel enzyme.

## Methods

### Isolation of CDA-producing organisms

Soil samples were collected from different ecological areas in north, central, and south China, including Chengdu, Shenyang, Xi’an, and numerous cities in Shandong province. The samples were screened for microbial strains with high CDA activity by diluting each soil sample (5 g) in sterilised water (50 mL) and incubating the sample at 37 °C for 30 min at 200 rpm. Each supernatant was then serially diluted and sprayed onto agar plates containing colloidal chitin as the carbon source and 4-nitroacetanilide (200 mg/L), which can easily penetrate bacterial cell walls and indicate deacetylation by changing colour. The plates were incubated at 37 °C for 2–3 days and desirable strains were screened based on the relative sizes of the yellow circles in the primary screen and using an enzymatic activity assay in the secondary screen.

### Culture conditions and phenotypic analysis of the strains

To allow fermentation, the strains were cultured in LB medium for 24 or 36 h at 37 °C and 200 rpm, after which their OD_600_ value and crude enzyme activity were measured, with each experiment performed in triplicate. The growth characteristics of each strain were determined using a Biolog system consisting of a microplate with 95 different carbon sources and a computer-driven automatic plate reader, wherein the four azole redox dye is reduced when a carbon source is consumed by the organism, causing a colour change from colourless to purple^35^.

### Scanning electron microscopy

SEM was performed as described previously^36^ with the following modifications. Briefly, the F6 strain was grown in LB medium at 37 °C for 24 h, centrifuged at 8000 r/min for 5 min, and the bacterial pellets washed five times with phosphate-buffered saline (PBS). The samples were fixed overnight using 2.5% (v/v) glutaraldehyde, washed thrice with PBS, and then covered with a gold layer prior to observation using an SU1510 FE-SEM (Hitachi, Japan).

### Phylogenetic analysis

The 16S rDNA of the identified candidate strain was amplified using universal primers (27 F and 1492 R) under the following colony PCR conditions: 94 °C for 5 min; 30 cycles of 94 °C for 30 s, 52 °C for 30 s, and 72 °C for 90 s; followed by 72 °C for 10 min. The purified PCR products were sequenced and their sequencing data subjected to phylogenetic analysis. The CDA amino acid sequences isolated from *Rhodococcus equi* F6 (ReCDA) were compared to those of known CDAs from other microorganisms^37–47^ by phylogenetic analysis, with a phylogenetic tree constructed using MEGA (version 7.0)^48^.

### Enzyme assays

The bacterial cells were washed, diluted with 0.2 M phosphate buffer (pH 7.0), and homogenised by grinding in a pestle and mortar in presence of liquid nitrogen. The enzymatic activity assay was performed as described previously^28^. Briefly, the reaction mixture was incubated at 37 °C for 1 h, with one unit of CDA activity defined as the amount of enzyme needed to catalyse the release of 4-nitroaniline per hour from 4-nitroacetanilide^28^.

### Analysis of polymeric chitosan and chitin deacetylation by crude *ReCDA*

To determine the ability of ReCDA to hydrolyse the acetamido group, we used a series of polymeric substrates, including chitin powder, colloidal chitin (prepared using chitin powder), chitosan polymers with 80 and 90% DD, and glycol-chitin (Wako, Japan). Crude ReCDA (5 mL) was stirred into an excess of substrate solution and the mixture was allowed to react for 12 h at 37 °C and pH 4.0 with agitation. Samples were then boiled for 5 min and the relative activity of the crude ReCDA toward different substrates was assessed by HPLC to measure the production of acetic acid^49,50^. Glycol-chitin and the product of glycol-chitin hydrolysis were also analysed by MALDI-TOF MS using an Ultraflex II TOF/TOF MALDI-TOF mass spectrometer (Bruker Daltonics, Germany) alongside an N_2_ laser with a 337-nm wavelength at a frequency of 50 Hz and positive ion-reflector mode at an accelerating voltage of 20 kV. All analytes were spotted on a 384-spot stainless steel plate and 2,5-dihydroxybenzoic acid (DHB) was used as the matrix, with 0.6 μL of matrix solution applied to each spot^29^.

### Whole genome sequencing, assembly, and annotation

The whole genome sequencing of *R. equi* F6 was performed by Suzhou Genewiz Biotechnology Co. Ltd. PCR products from *R. equi* F6 were cleaned up and validated using an Agilent 2100 Bioanalyser (Agilent Technologies, Palo Alto, CA, USA) and quantified using a Qubit 3.0 Fluorometer (Invitrogen, Carlsbad, CA, USA). Libraries with different indices were multiplexed and loaded using an Illumina HiSeq instrument (Illumina, San Diego, CA, USA), according to the manufacturer’s instructions. Sequencing was carried out using a 2 × 150 paired-end (PE) configuration, while image analysis and base calling were conducted using HiSeq Control Software (HCS)+OLB+GAPipeline-1.6 (Illumina) on a HiSeq instrument (Illumina). The library was also sequenced on a PacBio RSII/Sequel SMRT instrument^51^. Coding genes were annotated using BLAST in the National Centre for Biotechnology Information (NCBI) NR database. Gene function was annotated using the GO database, while pathways were annotated using the KEGG database. Proteins were classified phylogenetically using the COG/KOG Clusters of Orthologous Groups (COG/KOG) database.

### Nucleotide sequence accession number

The nucleotide sequence of *R. equi* F6 was deposited in the NCBI SRA database under the accession number PRJNA526377.

## Supplementary information

Supplementary Information.
