# Supplementary material for: Isolation, characterisation, and genome sequencing of Rhodococcus equi: a novel strain producing chitin deacetylase
Source: Sci Rep. 2020 Mar 9;10:4329. doi: 10.1038/s41598-020-61349-9 (PMC7062688; doi:10.1038/s41598-020-61349-9)
Supplement: Supplementary file 1 — Supplementary Information. [file 41598_2020_61349_MOESM1_ESM.pdf]

Isolation, characterisation, and genome sequencing of *Rhodococcus equi*: a novel strain producing chitin deacetylase

Qinyuan Ma[1#], Xiuzhen Gao[2#], Xinyu Bi[1], Linna Tu[1], Menglei Xia[1], Yanbing Shen[1*], Min Wang[1*]

[1]*Key Laboratory of Industrial Fermentation Microbiology (Tianjin University of Science & Technology), Ministry of Education, Tianjin Key Lab of Industrial Microbiology, College of Biotechnology, Tianjin University of Science and Technology, Tianjin 300457, P. R. China*

[2]*School of Life Science, Shandong University of Technology, Zibo 255049, China*

[#]These authors contributed equally to this work.

[*]Corresponding authors:

Min Wang: Tel.: 086 22 60600045; Fax: 086 22 60602298; E-mail: minw@tust.edu.cn

Yanbing Shen: Tel.: 086 22 60600045; Fax: 086 22 60602298; E-mail: shenyb@tust.edu.cn

Supplementary Table S1

CDA enzymatic activities of different colonies

Soil sample collection sites	Strain number	Primary screen (Relative size of the yellow circle)	Secondary screen (Enzymatic activity, U/ml)
Under trees in Fujia village, Fengjia town, Zhanhua county, Binzhou city, Shandong province, China	A1	+	4.19 ± 0.42
	A10	++	8.29 ± 0.69
	A21	+	3.22 ± 0.36
A small bamboo forests in Xiazhuang town, Rongcheng city, Weihai, Shandong province, China	F2	+++	18.28 ± 1.75
	F3	+++	16.37 ± 1.03
	F6	+++	36.22 ± 3.72
	F23	+++	31.03 ± 2.91
	F26	+++	27.63 ± 2.22

Under the piers of trestle, Yantai city, Shandong province, China	H13	++	1.26 ± 0.27
Beside a river, Yandian town, Yanzhou city, Shandong province, China	I14	++	6.27 ± 0.69
Under a tree in the park of Northeastern University in Shenyang, Liaoning province, China	J2	++	5.28 ± 0.44
In flowers on both sides of Yantai road in Xi 'an, Shanxi, China	M1	++	5.86 ± 0.83
	M4	+++	14.27 ± 1.52
	M6	+++	16.03 ± 0.96
	M7	+++	23.37 ± 3.10
In the woods on campus of Southwest Jiaotong University, No. 999 Xixian'an road, Danan county, Chengdu city, Sichuan province, China	O	+	4.21 ± 0.57

+, Weakly positive; ++, Positive; +++, Strongly positive

Supplementary Table S2

Biochemical and physiological characteristics comparison of strain F6 with some previously described *Rhodococcus* strains

Rhodococcus species	*R.tukisamuensis* Mb8^T	*R.jostii* IFO 16295^T	*R. pyridinivorans* PDB9^T	*R.maanshanensis* M712^T	*R.koreensis* DNP505^T	*R.zopfi* TI^T	Strain F6
Characteristic							
Colonies	Cream	light pink	light orange	cream	cream	red to pink	light pink
	opaque	opaque	opaque	ND	opaque	ND	opaque
	irregular edges	irregular edges	irregular edges and wrinkles	irregular edges	irregular edges	wrinkled	smooth
Cells	rod-shaped to cocci	irregular rods to short rods or cocci	rods to short rods or cocci	rods to cocci	rods to short rods or cocci	rods to irregular rods or cocci	Distinct cocci
pH	5.5-8.5	ND	6.0-9.0	6.0-8.5	6.0-8.0	ND	4.0-9.0
Temperature	15-45℃	15-30℃	30-37℃	25-30℃	25-30℃	ND	25-40℃

Utilization as sole carbon and energy source:							
D-Ribose	W	-	+	+	ND	ND	+
D-Fructose	W	ND	+	ND	ND	ND	-
D-Glucose	+	ND	-	ND	ND	ND	+
Sucrose	+	-	ND	+	ND	-	-
D-Mannitol	-	ND	+	-	ND	-	-
D-Sorbitol	-	ND	+	-	ND	-	-
D-Cellobiose	+	ND	ND	-	ND	ND	-
D-Arabinose	-	ND	ND	ND	ND	ND	-
D-Xylose	-	ND	-	-	+	ND	+
D-Galactose	+	ND	ND	+	+	ND	-
L-Rhamnose	+	ND	ND	W	+	-	-
Lactose	-	ND	ND	-	+	ND	-
Maltose	+	ND	-	+	+	+	-
Melezitose	+	ND	ND	ND	+	ND	-
D-Turanose	+	ND	ND	+	+	-	-
Arabitol	-	ND	ND	ND	+	ND	-
myo-Inositol	-	ND	ND	-	+	-	-
Xylitol	+	ND	ND	ND	+	ND	-
Inulin	+	ND	ND	-	+	ND	-
Tween 80	+	-	+	ND	ND	ND	+
N-Acetylglucosamine	ND	+	ND	ND	ND	ND	-
Glycerol	ND	ND	+	+	ND	+	+

References	16	17	18	19	20	21	This work

+, Positive; -, negative; W, weakly positive; ND, not determined.

Supplementary Fig. S1

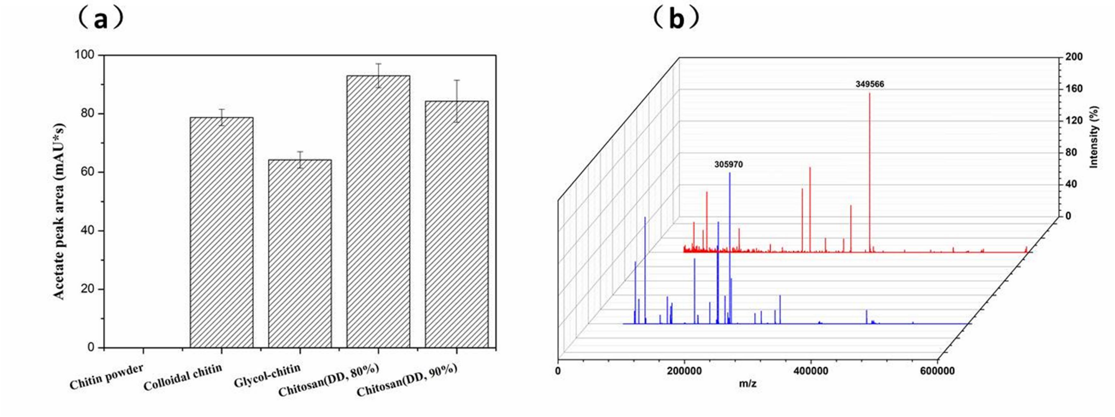

Deacetylation of polymeric chitosans and chitin by crude ReCDA. (a) Acetic acid levels detected by HPLC; (b) MALDI-TOF MS analysis of glycol-chitin and end product hydrolysed by ReCDA.

Supplementary Fig. S2

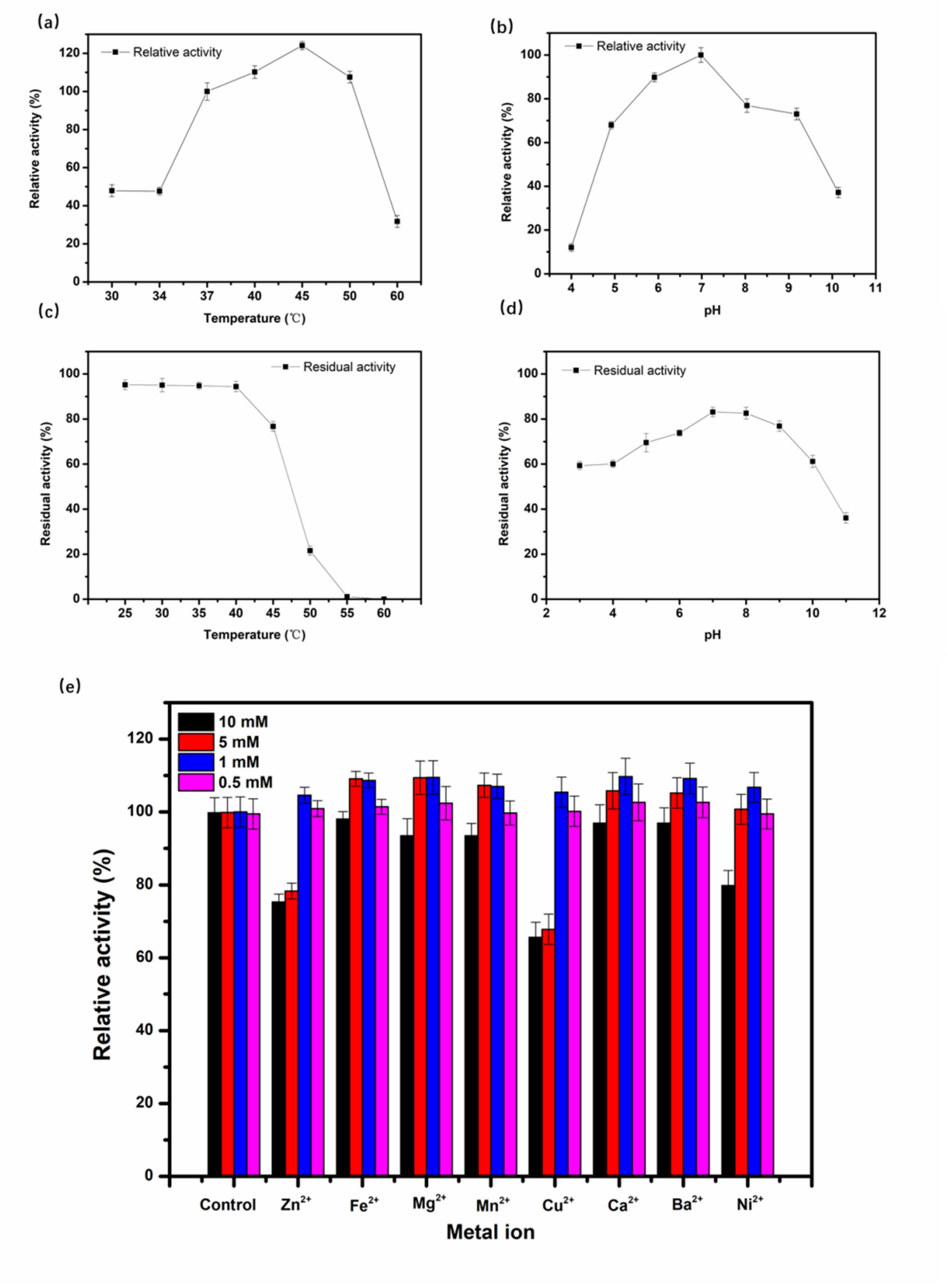

Biochemical properties of ReCDA. (a) Effect of temperature (30 °C to 60 °C) on enzymatic activity; (b) Effect of pH (4.0 to 10.0) on enzymatic activity; (c) Effect of temperature on the stability of ReCDA; (d) Effect of pH on the stability of ReCDA; (e) effect of metal ions on enzymatic activity.

To determine the optimum temperature, enzyme reaction was controlled under various temperatures (30-60 °C) for 60min. For the temperature stability, enzyme was pre-incubated at various

temperatures (25-60 °C) for 30 min without substrate. To determine the optimum pH, the enzyme reaction was performed in 0.2 M hydrogen phosphate-citric acid buffer (pH 4.0-8.0), 1/15 M phosphate buffer (pH 6.0-10) for 60 min. For the pH stability, the enzyme was pre-incubated in the various pH buffers at 4°C overnight [5]. For the effects of metal ions, the enzyme reaction was carried out in 0.2 M phosphate buffer (pH 7.0) mixed with different metal ions such as Zn^{2+}, Fe^{2+}, Mg^{2+}, Mn^{2+}, Cu^{2+}, Ca^{2+}, Ni^{2+} (0.5-10 mM) [6]. The enzyme activity (157.6U/mL) detected at 37 °C and pH 7.0 without incubation was used as the standard (100%) to calculate the relative and residual activities.

Supplementary data 5

The gene sequence of ReCDA

ATGTCCAAGAAGATCTACGTCAACTTCGGTGTCGACGTCGATGCCGTCGCCGGCTGGCTCG

GGTCGTACGGAGGCGAGGACTCGCCCGGCGACATCTCCCGCGGCCTCTTCGCAGGAGAGG

TCGGCGTTCCGCGGCTGGTCAAGCTGTTCGAGAAGTACGGCATCACGACGTCCTGGTTCGT

GCCCGGCCACTCGATCGAGACGTTCCCGCGCGAGATCGAGCAGGTAGTCGCGGCCGGCCA

CGAGATCGGCATCCACGGCTACAGCCACGAGAACCCGATCGCGATGACGCGTGAGCAGGA

GACCGCGGTCCTCGACCGCTGCATCGAACTGATCGAGTCCTTCACGGGTCGCAAGCCCACC

GGTTACACGGCACCGTGGTGGGAGTTCTCCAAGGTCACCAACGAACTGCTGGTCGAGCGC

GGCATCAAGTACGACCACTCGCTCATGCACAACGACTTCACGCCCTACTACGTCCGTGTCG

GCGACTCCTGGACCAAGATCGACTACAGCCAGCCGGCCGAGACCTGGATGAAGCCGCTCG

AGCGTGGCGCGGAGACCGACCTGGTCGAGATTCCCGCGAACTGGTTGCTCGACGATCTGC

CGCCGCAGATGTTCATCAAGTCCAGCCCCAACAGCCACGGTTTCGTCAGCCCGCGGCATCT

CGAGGAGATGTGGCGCGACCAGTTCGACTGGGTCTACCGCGAGATGGACTATGCGATCTTC

CCGATCACCATCCATCCCGACGTGTCCGGCCGTCCGCAGTCCCTGCTCATGCTCGAGCGCCT

CATCGAGCACATCAACAAGCACGACGGGGTCGAGTGGGTCACGTTCGACCAGATGGCCGA

CGACTTCAAGGAACGGTTCCCGCGCCGGAACTGA

Supplementary Table S3

Chitin degrading enzymes in *R. equi* F6 identified by the CAZy database

Function	Enzymes	Substrate	CAZy family	Number of genes
Deacetylase	Chitin deacetylase	Chitin, Chitooligosaccharide, Peptidoglycan, Acetyl xylan	CE4	1
	N-acetylglucosamine 6-phosphate deacetylase	N-acetylglucosamine 6-phosphate	CE9	2
	N-acetylglucosamine deacetylase	N-acetylglucosamine	CE11	4
	Diacetylchitobiose deacetylase	Diacetylchitobiose	CE14	16
Hydrolase	Chitinases of classes III and V	Chitin	GH18	1
	Chitinases of classes I, II, and IV	Chitin	GH19	3
	Modules of approx for chitinases	Chitin	CBM12	6
	Modules of approx for chitinases	Chitin	CBM14	3
	Modules of approx for chitinases	Chitin, Peptidoglycan	CBM50	15
